# The Role of Health Co-Benefits in the Development of Australian Climate Change Mitigation Policies

**DOI:** 10.3390/ijerph13090927

**Published:** 2016-09-20

**Authors:** Annabelle Workman, Grant Blashki, David Karoly, John Wiseman

**Affiliations:** 1 EU Centre on Shared Complex Challenges, Australian-German Climate and Energy College, The University of Melbourne, Melbourne 3010, Australia; 2 The Nossal Institute for Global Health, The University of Melbourne, Melbourne 3010, Australia; gblashki@unimelb.edu.au; 3 School of Earth Sciences, EU Centre on Shared Complex Challenges, The University of Melbourne, Melbourne 3010, Australia; dkaroly@unimelb.edu.au; 4 Melbourne Sustainable Society Institute, Australian-German Climate and Energy College, The University of Melbourne, Melbourne 3010, Australia; jwiseman@unimelb.edu.au

**Keywords:** climate change, mitigation policy, Australia, health, co-benefits

## Abstract

Reducing domestic carbon dioxide and other associated emissions can lead to short-term, localized health benefits. Quantifying and incorporating these health co-benefits into the development of national climate change mitigation policies may facilitate the adoption of stronger policies. There is, however, a dearth of research exploring the role of health co-benefits on the development of such policies. To address this knowledge gap, research was conducted in Australia involving the analysis of several data sources, including interviews carried out with Australian federal government employees directly involved in the development of mitigation policies. The resulting case study determined that, in Australia, health co-benefits play a minimal role in the development of climate change mitigation policies. Several factors influence the extent to which health co-benefits inform the development of mitigation policies. Understanding these factors may help to increase the political utility of future health co-benefits studies.

## 1. Introduction

The twenty-first United Nations Framework Convention on Climate Change (UNFCCC) Conference of Parties (COP21), held in Paris in December 2015, has been heralded as a key milestone in global climate change negotiations [1]. In the days following the conclusion of COP21, United Nations Secretary-General Ban Ki-moon asserted that the resulting climate accord, the Paris Agreement, represented a ‘health insurance policy for the planet’ [2]. Despite widespread enthusiasm for the achievements of COP21, there is also broad acknowledgement that, in combination, the Intended Nationally Determined Contributions (INDCs) submitted by Parties to the UNFCCC prior to COP21 fall well short of the agreed pledge in Paris to limit global temperature rise to well below two degrees Celsius above pre-industrial temperatures. UNFCCC estimates suggest current INDC pledges will lead to a temperature increase of approximately 2.7 degrees by 2100 [3]. This dilemma reinforces findings in the latest Intergovernmental Panel on Climate Change (IPCC) report. The IPCC’s Fifth Assessment Report Synthesis Report asserts that action on climate change is not commensurate to the severity and likelihood of anticipated impacts, given “global increases in anthropogenic emissions and climate impacts have occurred, even while mitigation activities have taken place in many parts of the world” [4] (p. 54).

The combustion of fossil fuels has both longer-term and shorter-term impacts on health. In the longer term, fossil fuel use contributes to climate change, with significant health impacts on populations. For example, it is anticipated that climate change will impact human health through increased frequency of extreme weather events and increased prevalence and distribution of infectious diseases [5,6,7]. In the shorter term, fossil fuel use creates air pollution through the release of particulates and climate altering pollutants. Both ambient and indoor air pollution can significantly affect respiratory and cardiovascular health outcomes for populations [8,9,10].

With this in mind, research has explored the role that health outcomes can play in providing a justification for enhanced domestic climate change action. Research undertaken around climate change communication has found that framing climate change from a health perspective can positively influence an individual’s acceptance of robust climate policies, irrespective of political preferences [11,12]. Further, the health co-benefits literature has established that there are significant, shorter-term, and localized health—and consequently economic—benefits that result from the implementation of emissions reduction (mitigation) policies [13]. For example, in justifying the implementation of the Clean Power Plan in 2015, the United States (US) Environmental Protection Agency estimated that, by 2030, health benefits for the US would include up to 3600 fewer premature deaths and up to 90,000 fewer pediatric asthma attacks, totaling an economic saving of up to USD$54 billion in 2030 alone [14]. These health co-benefits have been quantified to allow for comparison with economic costs often considered in the development of climate change mitigation policies. While environmental health impacts researchers acknowledge that health co-benefits have not gained the political traction they potentially warrant in the development of climate policy [15,16,17], there is minimal research examining the role of health co-benefits in the development of national climate change mitigation policies. To begin to address this knowledge gap, we have explored how health co-benefits have been considered and accounted for in the development of Australian climate change mitigation policies.

Australia provides an interesting case study for exploring the role of health co-benefits in the development of national mitigation policies. Economically, its strong and influential fossil fuel resource sector significantly contributes to gross domestic product. Politically, climate change has proven to be a polarizing and controversial policy area, implicated in several political leadership changes and federal election results [18,19]. Australia is the first country to repeal a carbon pricing mechanism, following the rise to power of the Coalition government in 2014. Instead of a price on carbon, the Coalition government has pursued a direct action approach to meet domestic and international carbon abatement targets, a scheme that involves government purchases of domestic abatement opportunities. Internationally and domestically, it has been described as being a laggard in its climate policy ambition [20]. In the lead-up to the most recent federal election, held in July 2016, polling suggested public support for action on climate change was the strongest it has been since 2008 [21]. As is explored in the case study below, these and other factors have influenced the Australian narrative on climate change, as well as the role and influence of multiple considerations, including health, in the development of Australian climate change mitigation policy.

## 2. Methods

**Figure 1:**
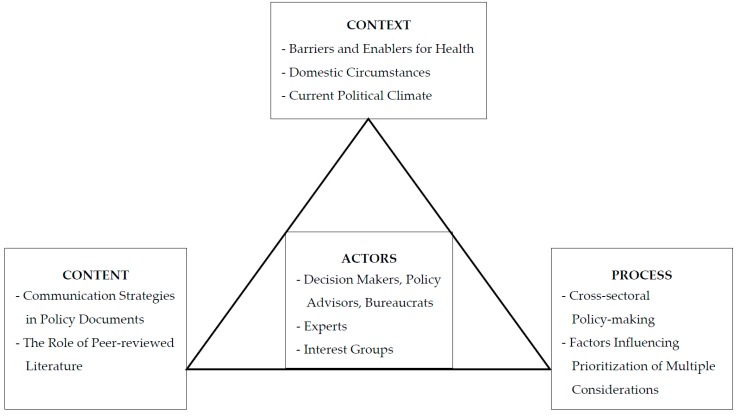
Modified from Walt and Gilson’s model for health policy analysis.

Theoretically, we situate this research within the political economy of health, which provides a robust framework in which to explore health within the climate change agenda. Health is inherently political in nature given “power is exercised over it as part of a wider economic, social and political system” [22] (p. 187). The political economy of health framework contends that both good and ill health are a “result of social, political, and economic structures and relations” [23] (p. 134) that can be easily overlooked given health is often defined in a functional and individualistic manner [24] (pp. 34–35). To support the application of this theoretical framework, a complementary analytical framework was identified and used to inform the research project. A previous analysis of health factors in climate change policy has been undertaken by Morrow and Bowen [25] who investigated the consideration of health in Fijian climate change policies. In line with their approach, the development of this case study is underpinned by Walt and Gilson’s [26] policy analysis framework (see Figure 1).

This model provides a holistic approach to the consideration of the policy-making process, by moving beyond purely content analysis to consider broader contextual and process factors that are likely to influence the policy development process. While the model above provides a simple, schematic representation of what are inherently complex relationships, Walt and Gilson assert it can be used to facilitate the exploration of policy as an “outcome of complex social, political and economic interactions” [26] (p. 359). With this in mind, key factors influencing the development of mitigation policy have been incorporated into our assessment of Australia’s mitigation policy development, including Australia’s politico-economic context; governance structures and policy processes; cultural factors and public attitudes toward climate change; the role of external actors and stakeholders; as well as the climate change narrative and drivers for mitigation policy communication.

### 2.1. Study Design

A case study approach was chosen to undertake this research, as it is the preferred approach when posing “how” and “why” questions in an attempt to “understand social complex phenomena” [27] (pp. 13–14). With Walt and Gilson’s model in mind, several data sources were used to develop a comprehensive Australian case study. Semi-structured interviews constitute the primary data source, and were undertaken with individuals who met the eligibility criteria: federal government employees involved in the development of mitigation policies. Interviews are supplemented by secondary sources, primarily key recent federal government policy documents (see Table 1) that were identified prior to and during interviews.

Feedback on the semi-structured interview schedule was sought from four academic experts and one environmental health policy expert; the policy expert provided feedback, suggesting minor changes, which were incorporated. The interview schedule comprises eight questions that fall into one of six key themes: the policy-making process; factors influencing the prioritization of multiple considerations; barriers and enablers for the consideration of health in mitigation policy; the evidence base for policy; the role of external actors and stakeholders; and the communication of policy decisions. The themes loosely correlate to one of the four elements identified in Walt and Gilson’s policy analysis model (see Figure 1). The interview schedule was submitted along with an ethics application to the University of Melbourne Faculty of Science Human Ethics Advisory Group. Ethics approval was obtained in November 2015 (Research Project 1545561.1).

### 2.2. Recruitment of Stakeholders

Given the political sensitivity that has surrounded Australian climate policy in recent years, we anticipated that the recruitment of federal government employees might be challenging. Consequently, we utilized personal networks and networking at conferences in the first instance followed by snowball sampling to recruit participants. We sought to recruit at least one employee who met the eligibility criteria outlined above from across six departments associated with the development of national energy- and transport-related mitigation policies (see Table 2). Permission was sought to record interviews to aid in the transcription process. No participants objected to the recording of their interview. Individuals who agreed to participate were informed that transcripts would be de-identified in order to protect their identity. Consent forms were received from all interviewees prior to interviews being conducted. We transcribed all interviews verbatim, and then verified all interview transcripts to ensure accuracy of the transcription process.

### 2.3. Data Collection

The interview schedule was piloted three times between April and May 2016 by participants with a background in either Australian health or climate change policy. Two of the three participants provided permission for their interview data to be incorporated into the case study. The interview schedule did not change between the pilot and formal phase of interviews. The first author conducted all formal interviews with federal government employees who met the eligibility criteria between June and July 2016. This period coincided with the Australian government assuming a caretaker role in the lead-up to the July 2016 federal election. In total, eighteen individuals were approached for interview. Four individuals declined, suggesting more appropriately placed colleagues to participate. Two individuals were non-responsive and one individual left their respective Department prior to the interview. Of the eleven interviews that eventuated, six interviews were carried out face-to-face in either workplaces (*n* = 5) or at neutral locations (*n* = 1), and five interviews were conducted by phone. Interview lengths ranged from 38 to 75 min.

Snowball sampling identified one additional individual who did not meet the eligibility criteria but who nevertheless had relevant expertise in the development of Australian climate change mitigation policies given a previous high-level position in federal climate change policy development. Interviews with this participant followed the same process as the formal interviews, and data from this interview informed the development of the case study. While the number of interviews carried out was effectively determined by time constraints, despite the small sample size, we assessed that data saturation was achieved as evidenced by the repetition of themes and a lack of new themes emerging.

### 2.4. Data Analysis

De-identified transcripts were imported into NVivo 11 and initially coded based on the themes identified in the interview schedule. Additional sub-themes were identified during the coding process and have been integrated into the results and discussion sections below.

### 2.5. Limitations

There are some limitations to the methodological approach utilized for this research. Firstly, as a matter of practicality, single coding of interviews was solely undertaken by the first author. Given coding was guided by the structure of the interview schedule, we do not consider this to be methodologically compromising.

Secondly, using a semi-structured interview schedule allowed a level of flexibility in question order based on the natural flow of the conversation; however, for most interviews, the question order primarily aligned with the interview schedule question order. This may have influenced responses and further discussion. For example, barriers to health considerations within climate policy were generally discussed before enablers, given a majority of interviewees openly acknowledged that health was not a significant consideration in the policy development process. It is possible that question order in this instance influenced the ability of interviewees to consider enablers.

Finally, conversation often led to discussion of co-benefits; discussion of potential health co-harms from the development of mitigation policy did not ensue. We acknowledge this as a limitation of the research, and suggest that future research explore the consideration of health co-harms in the development of climate change mitigation policy.

## 3. Results

The analysis of interviews and secondary sources provides a level of insight into the role of health co-benefits as a consideration in the development of Australian climate change mitigation policy. The results are presented below in line with the themes used during the interview schedule. We elaborate on sub-themes where they have been identified during the coding process.

### 3.1. Policy-Making Process

Most interviews began with broad policy-making discussions, exploring the processes used to account for multiple considerations in cross-sectoral policy areas, and how who is “at the table” is determined. Most interviewees outlined the whole of the government approach that is used at the federal level to develop cross-sectoral policies. Interviewees described the cabinet submission development process. In line with Australian Administrative Orders, the Department of the Environment is the line (or central) agency for domestic climate change mitigation policy, while the Department of Foreign Affairs and Trade was primarily responsible for the development of Australia’s INDC that was taken to COP21. To inform the Cabinet submission, the line agency may decide to establish an interdepartmental committee (IDC) for the purpose of seeking input from other relevant agencies. A regulatory impact statement (RIS)—a form of impact assessment—would generally be included as part of the cabinet submission process, and may be accompanied by a cost–benefit analysis. The Office of Best Practice Regulation (OBPR) was identified as the gatekeeper of RIS development, responsible for determining the robustness of the quantitative data underpinning a RIS, as well as RIS approval. Interviewees noted that, irrespective of an IDC, all departments are provided an opportunity to provide input, comment, or both on each cabinet submission prior to its consideration by cabinet ministers. Many interviewees emphasized that quantifying and monetizing multiple considerations, particularly costs, constituted an integral component of the policy development process:

“…what we’re encouraged to do as often as we can is to monetize things, not necessarily because money is how the world goes round, but because money is a common um, it’s a common way of measuring things…we’re often encouraged to do an economic analysis because what it does is it allows us to compare otherwise quite disparate things…”

(I_01)

A number of interviewees also made the distinction between quantifying costs and benefits, and how this impacts upon their consideration in a RIS:

“…usually you can quantify the costs relatively well…and then, you can usually quantify some benefits relatively easily but then there tends to be a whole class of benefits that are difficult to quantify, and what you will often do in a regulatory impact statement is reference them qualitatively but not try and quantitatively [value] them…Now sometimes that would be because your benefits already exceed your costs, so you can consider them upside, but other times that’s purely because you don’t have the data, and if you try to put the case up, then, you know, if you, if you depend on that and you don’t, you don’t have enough actual support, then it could undermine your policy because, you know, it’s a piece, it’s like better not to…depend on something if you can’t defend it.”

(I_03)

A number of interviewees noted that the Department of Health would not have been considered one of the core agencies during the development of mitigation policy. For example, while the Department of Health did provide comment on the proposed INDC that was taken to cabinet in the lead-up to COP21, several interviewees from across departments stated that they were a peripheral agency in the target development process:

“…so…when we convened IDCs invited health, um, ah, to those meetings, and the comments that health made at the time, you know, they were engaged, which was um, good, but they were very generalized statements…”

(I_04)

### 3.2. Factors Influencing the Prioritization of Multiple Considerations

Interviewees were asked to comment on the different processes used to rank or prioritize different considerations that inform policy development. Economic and employment considerations were primarily discussed as informing the development of mitigation policy in Australia. Several factors will influence the extent to which these considerations are prioritized in the development of mitigation policies, although bureaucrats do not overtly rank or prioritize considerations themselves:

“…we didn’t rank particular um, aspects as more important than others in, in the decision-making process, like we didn’t rank economic over um, social well-being…or the climate impacts. Um, so, so we didn’t but you can bet that the people making the decisions were weighing those things up in their minds and assigned different values to them.”

(I_10)

One factor influencing the prioritization of multiple considerations is that ministers are individually responsible for agenda setting within their own department or portfolio, which inevitably informs the direction of policy development, and the consideration and prioritization of multiple considerations:

“…there are a number of…criteria the government use. How they actually in the end come up with that is, is, is hard to distinguish, so each, each minister and each, each portfolio would come to it with their own ah, priorities, set of priorities…we’re really focused on making sure that we are…we, we know exactly what the rest of the world is doing, and that we are, you know, we’re in the pack and…we compare well to the rest of the world essentially. Um, Treasury will have a different view. Um, Environment will, you know, want to know that whatever policies we set we can meet domestically…”

(I_02)

The political reality of Australian climate change policy is another factor that influences how considerations are prioritized in the development of mitigation policy. A number of interviewees conceded that the politicized nature of the climate change debate in Australia has in part determined which considerations are included in the development of mitigation policy, and how these considerations influence policy development:

“…it becomes a political judgment amongst the policy-maker essentially about um, how much pain am I going to suffer as a result of choosing a particular outcome and because the current environment of climate policy in Australia is so politically toxic it makes them, everyone risk averse”

(PI_02)

“…the problem Australia’s had…is just how toxic the debate has been and how politicized the debate has been, and therefore it has been hard to have that considered um, there hasn’t been the bandwidth to have that kind of conversation with the public about this.”

(I_02)

The core climate change narrative in Australia is a third factor that influences which and how multiple considerations are prioritized. Almost all interviewees acknowledged the crucial role that economic considerations play during the development of mitigation policies, asserting that economic factors are always first order. Economic analysis or modeling, or both, is often used to inform the development of policy and can strongly influence the government’s priorities and choices. This is in part explained by the relative ease of quantifying the impacts, particularly costs, to the economy of mitigation action. However, it can also be in part explained by the government’s current narrative on climate change, which according to some interviewees, frames climate change action as an economic burden with the potential to create issues for the competitiveness of major industries:

“…it really depends a bit on the government of the day. I mean the government we had last year was about um…economic growth and, and jobs and preserving industry and making sure we do what the rest of the world’s doing and you know, a range of those factors…”

(I_02)

“…if you read for example, the um, issues paper produced by PM & C last year before the decision was taken on what Australia’s 2030 target should be…if you read that, you’ll see it’s still, you know, all about burdens and competitiveness, and if we cut back, if we put in a carbon price, you know, what happens to our aluminium sector…when others don’t do it, leakage, all that sort of stuff, that’s all the old argument.”

(I_07)

The UNFCCC Taskforce final report [29] (p. 21) mentions that, during the submission process, some individual submissions highlighted the ‘consequences of inaction, such as environmental and health impacts…’, but there is no indication of whether or how these impacts were taken into account during the INDC target setting process. A direct query about this during one of the interviews elicited the following response:

“I think that the um, to the extent, things like health were factored in, it was this general ah, vibe if you like, of um, there’s a cost of not doing anything, um, and it was, and that um, in my view, wasn’t a particularly strong factor and it certainly wasn’t a ah, a consideration that was unpacked in a very detailed and systematic way, it was just a, as I say a general thing of, there are costs of not taking action.”

(I_04)

In relation to the consideration of health, interviewees determined that it is currently a second- or third-order issue, similar to other sectors of the economy that are inevitably affected by climate change but do not significantly influence policy decisions:

“I think at the moment health is seen as…relevant to climate change in the same way that infrastructure, and you know, numerous other things are, and they’re all grouped together in this sort of, climate change is going to have broad impacts across the whole scope of our economy and public policy…so there just becomes this sort of homogenous mass of stuff…”

(I_04)

The de-prioritization of health as a potential co-benefit of mitigation measures becomes evident when analyzing key policy documents, such as the Emissions Reduction Fund White Paper [28] (p. 7):

“The Emissions Reduction Fund will help reduce Australia’s greenhouse gas emissions while delivering valuable co-benefits to Australian businesses, households and the environment. For example, households and businesses will save money by improving their energy efficiency. Revegetation will improve water quality, and reduce erosion and salinity. Replenishing the carbon content of soils will improve the health and productivity of Australian farms.”

As one interviewee noted when discussing the Emissions Reduction Fund White Paper:

“…what’s useful to look at is the communications…you’ll often see phrases along the lines of um, um, this policy is reducing emissions while um, improving the productivity of farms, cutting costs, and um, increasing the productivity of, of businesses…you can see that what, what is being done there is very overtly talking up the co-benefits as a way of saying this is a great policy and it’s ticking lots of the boxes…Now there’s no obvious reason why, why health benefits couldn’t be included in that, in that list of dot points…in this instance, we, you know, at the moment we talk up the productivity or economic benefits…”

(I_01)

The same government rhetoric emphasizing reduced emissions while improving productivity and competitiveness is evident in Australia’s National Energy Productivity Plan 2015–2030 [31] (p. 6):

“By increasing our energy productivity we strengthen our economy and help safeguard our environment. Businesses reduce their energy costs through innovation and modernizing their infrastructure—improving their output and making them more competitive. Household consumers benefit through lower energy bills and increased home comfort. At the same time, Australia reduces its carbon footprint and contributes to the global challenge of mitigating climate change. It’s a win, win, win for Australia.”

### 3.3. Barriers and Enablers for the Consideration of Health in Mitigation Policy

#### 3.3.1. Barriers

Interviewees were asked what they considered to be potential barriers and enablers in accounting for health in the development of mitigation policies. Several barriers that impact the consideration of health co-benefits in the development of climate change mitigation policy were identified.

These fall into two broad areas:

1. A lack of expertise within government, advocates outside of government, and context-specific robust data; and
2. The long-term nature of health impacts, the shorter-term issue of an “invisible” problem, the challenges of distinguishing and articulating the link between the combustion of fossil fuels and health impacts, and the primary consideration of health within climate change adaptation policy.

In the first area, interviewees identified a lack of strong advocacy from within and outside of government for the inclusion of health co-benefits in the development of mitigation policy. A number of interviewees acknowledged that federal government employees tasked with the scoping and development of mitigation policy were unlikely to have a health background and relied on the Department of Health to provide relevant input:

“…it’s not this department’s, it’s not PM & C’s, it’s not DFAT’s job to understand the health impacts of climate change, it’s the Health Department’s job to bring those considerations to bear, and so it kind of depends on them prioritizing it and having the capability around, around that function.”

(I_04)

Interviewees also raised the issue that the number and prominence of Australian climate change and health experts and advocates from the health sphere presented a challenge. The late Tony McMichael, a leading Australian epidemiologist and environmental health expert, was acknowledged as a well-regarded Australian climate change and health expert with a level of influence. However, some interviewees felt that there were now few resounding academic champions on the issue of climate change and health within Australia, and those who were in the space were yet to genuinely capture the government’s attention:

“…a key actor in the field, like um…Anthony McMichael was massive in his day…we worked quite a lot with him, so if someone wanted to get to us, they’d go through him and then he’d raise it with us and then that would be taken notice of…”

(I_09)

“…yes you have, you know, a few, a few very visible and, and expert ah, public health officials talking about the climate in public, ah the climate and health debate, but you don’t have them um, linking that to the core government narrative on climate change…that’s one about the economics, it’s around what other countries are doing, and essentially I think you want to flip it from being a defensive and problematic issue to an opportunity issue…”

(I_02)

In addition, interviewees conveyed that the lack of local, robust evidence inhibited the inclusion of health co-benefits in policy development in any meaningful way. It was recognized that, while health co-benefits had the potential to be used to bolster the rationale for ambitious action, in the absence of a defensible evidence base situated within the Australian context, the inclusion of health co-benefits as a consideration in mitigation policy may actually undermine any policy proposal put forward to cabinet:

“…the data, quality data just doesn’t seem to be in existence, particularly for Australia…there’s stuff out of the US and the EU, ah, and all that data is done in a contextual environment, bigger cities, different weather conditions, all those sorts of things, so it’s not directly translatable to Australia necessarily.”

(I_05)

“…if it’s not strongly defendable or robust data, it comes under criticism, undermines a whole lot of the argument, not just the health bit of the argument…”

(I_05)

In the second area, interviewees identified the conundrum of longer-term health impacts from climate change and the challenge of drawing clear, defensible links between health co-benefits, climate change, and the combustion of fossil fuels. While some interviewees were able to articulate the distinction between the longer-term health co-benefits associated with climate change and the shorter-term health co-benefits associated with the mitigation of fossil fuel use, some interviewees found it difficult to acknowledge that Australia would see any domestic health gains from the implementation mitigation measures, reiterating that avoided health costs from climate change would only result from concerted global effort to address climate change:

“…yes it’s true that if you cleaned up the ah, if you reduced emissions in the, in the Latrobe Valley it would also clean up the Latrobe Valley, but the materiality of these things is just very different from, I mean you just have to go to Beijing to realize that um, quite apart from global warming they’ve got to do something about the smog in Beijing and that’s true of lots of big um, big ah, Chinese cities, so um, it, it absolutely makes sense from their point of view to talk about the co-benefits. That absolutely makes sense, but I don’t think it makes anything like the same amount of sense, for the sort of things we’re going to do to reduce emissions, ah, changing the source, changing the um, the energy mix that goes into electricity generation, making fuel, cars more fuel efficient, you think about the various things we’re going to do…there may be co-benefits but they’re going to be tiny by comparison with other countries…”

(I_06)

Interviewees suggested that the longer-term impacts to health from climate change increase the challenge of considering health during the development of mitigation policies:

“…so the government line is that because there’s no sort of direct links with um, well there are links, there are actually inalienable links, links between climate change and health, but you, you can’t put it down on paper and say this, this degree of change in heat will definitely arrange in this sort of um, illness or that sort of thing…I do think it generally acts as a barrier but I don’t think that’s anyone’s fault, I just think it’s the nature of the game because it’s all, it’s all concomitant and variation so, it’s all a case of, you know, there’s a change in the climate, and then there’s a corresponding change in…prevalence of respiratory diseases and only then do you get the corresponding change of…health, health is sort of at the bottom. And it’s, there’s so many, there’s so many easy ways to break the links between the two that…you’re never going to get anyone to agree that climate change is to, is to um, is to blame.”

(I_09)

“…when people say you die of a heat wave…a lot of people don’t associate it with, you know, their, their gran had a heart attack. They thought she was old, she had a heart attack…”

(I_03)

There was also an acknowledgement from some interviewees that competing priorities, particularly in the health domain, exacerbate the de-prioritization of health co-benefits:

“…you go to the health department and it’s not their biggest issue, right, it’s their fiftieth issue. And you go to the local government, and it may not be their biggest issue, it’s their fiftieth issue…”

(I_03)

“…who’s got the most pressure on which particular areas, I mean, that’s why in health in many ways, treatment is so much easier than prevention…you can’t make money in prevention, I mean you can, you know, you lift taxes, you know, sure, but that’s not, that’s not intrinsically the prevention industry producing that…”

(PI_01)

Finally, numerous interviewees spoke about health’s inclusion within the realm of climate change adaptation policy:

“…I think, within public policy in, in the Australian public service, the extent to which health is relevant to climate change is seen through an adaptation lens primarily, not through a mitigation lens.”

(I_04)

“ …I’ve done a little bit of work in adaptation, only, only bits and pieces, briefly, and a lot of health issues obviously are in the adaptation rather than the mitigation side…”

(I_03)

“…in terms of making decisions about the target, I don’t think other than as one of the many things that adds up, um, health played a big part. Where we see most of its activity is more kind of in that, that adaptation side.”

(I_10)

Health as a key focus of adaptation policy is reiterated in the Australian government’s National Climate Resilience and Adaptation Strategy [30] (pp. 58–59):

“…climate change poses challenges to the health of Australians through stresses such as heatwaves, droughts and an increase risk of food and water borne diseases. …Australia is responding to the health effects of climate change within the overall context of existing health services and the preventive health mechanisms that help provide a healthy and safe environment—for example, clean water and air, safe food and housing, and protection from pollutants and the spread of disease. State and territory governments play a crucial role in delivering health services across Australia…”

Of note, cross-jurisdictional governance structures within Australia see health and adaptation policy primarily the responsibility of state and local governments. While the inclusion of health in adaptation policy was not explicitly discussed with interviewees as a potential barrier to its consideration in mitigation policy development, the statement above from the National Climate Resilience and Adaptation Strategy reinforces that positioning health as an adaptation issue facilitates the transfer of responsibility for health in the climate change agenda from the federal government to state and local governments. This inevitably acts as a barrier to any meaningful consideration of health in national climate change mitigation policy development.

#### 3.3.2. Enablers

Interviewees found it difficult to identify current enablers for the consideration of health co-benefits in the development of climate change mitigation policies. A number of potential or prospective enablers were identified, but these were primarily based around a visible increase in impacts over the coming decades and decreasing technological costs in the energy and transport sectors. While not necessarily pertinent to national policy, one interviewee raised the recent Hazelwood coalmine fire in the state of Victoria as a potential enabler for increasing the role of health co-benefits in the development of mitigation policy:

“…It will be fascinating to see what happens with the Latrobe Valley post the Hazelwood mine fire…if you’re a politician who needs to make a decision about closing a coal-fired generator on the back of something like that happening…and there’s people dying from coal pollution, it makes your job a hell of a lot easier…”

(PI_02)

Opportunities to increase the role of health co-benefits in the development of mitigation policy were discussed during interviews, and are outlined in further detail in the Discussion section below.

### 3.4. The Evidence Base for Policy Development

Interviewees were asked about the extent to which peer-reviewed scientific literature is used in the development of policy, in order to determine whether the health co-benefits literature may have the potential to inform mitigation policy development. Opinions varied on the importance and inclusion of peer-reviewed literature in the policy development process. Most interviewees felt that peer-reviewed literature was considered to some extent in policy development; however, accessibility issues at times presented a challenge to its consideration and inclusion. In the absence of good quality domestic research, interviewees indicated that international research from reputable organizations and agencies, such as the International Energy Agency or the Organization for Economic Cooperation and Development, was also considered and utilized during policy development.

Beyond the peer-reviewed literature, interviewees indicated that synthesized information products from domestic think tanks and institutes were often useful and included in ministerial briefings or policy documents. There was also recognition that time constraints were imperative for the consideration of research and peer-reviewed literature:

“…it takes sometimes a while for peer-reviewed literature to get out, and sometimes you want a quick answer, and I sometimes say that…there’s either a three-minute answer, a three-month answer or a three-year answer, and you’ve got to be really clear about, you know, what you’re looking for…”

(I_02)

Many interviewees highlighted that relevant experts and peer-reviewed authors at times provided direct input into policy development. There was an acknowledgement that experts represented one group of key stakeholders in the policy process (discussed further below); however, their level of influence on the policy decisions was relatively limited due to structural and communication issues:

“…the kind of incentives and milestones that are placed on academics are very, unique um, and the sort of timeframes that I have on things are also very unique, and…people in the academic world wouldn’t know what I’m working on until later, but, I mean, what, what we’ve tended to is you know, find academics who are particularly relevant to us, and become really good mates with them. Um, so I have had academics who’ve had a lot of influence over what we’re doing, but regularly, they’re a subset of the academics that could be influencing us…”

(I_03)

“I think academics um…at least some of them that I’ve spoken to especially recently seem to expect that public servants will have the same sort of depth of um, understanding and analytical rigor as, as them, um, which we don’t, like we’re not, we’re not academics um, and so what we actually need is for academics to understand that we’re different and, um, to, there’s a bit of a, it’s almost a language barrier between, you know, academic speak and public policy speak…”

(I_04)

### 3.5. The Role of External Actors and Stakeholders

Interviewees were asked in what ways external actors and stakeholders inform the policy-making process, and whether there are avenues beyond the formal consultation processes that facilitate stakeholder input. Three key groups of stakeholders relevant to the development of Australian mitigation policy were discussed: business and industry stakeholders; non-governmental organizations (NGOs); and experts. The role of community stakeholders and public attitudes were touched on briefly by some interviewees, but did not provide any real insight into their role in policy development. Broadly, there was recognition of the importance of stakeholders in the political process:

“…in government you need to have stakeholders and you need to know who’s out in the field, and you need to be well-networked…ministers and minister’s offices ah, have meetings with these people, and they, and then they ask us to come along and, or give briefing, and or say, we’ve just met with this person, you know, and it filters down to us to, to action it. Or it’s, as I said earlier, us through our network, gathering those ideas, and, part of that is self-preservation for bureaucrats because it’s a contestable space and if we’re not providing advice, they’ll go, the government will go looking for it elsewhere…”

(I_02)

In relation to formal consultation processes, many interviewees indicated that discussion and other government policy papers released for consultation would likely have already had a level of input through targeted consultations and direct engagement with a number of influential and relevant external actors and stakeholders. It was recognized that there are resource limitations which impact the amount of consultation that is undertaken, but also that the process can become less valuable over time as the same issues are raised time and time again. Many interviewees also noted that informal processes tended to provide a greater level of influence than formal processes:

“…direct engagement with policy-makers, be they at the political level or the bureaucratic level is probably um, as influential if not more influential than the formal um, public submission processes…”

(I_04)

A number of interviewees accepted that business and industry stakeholders are the most influential in the policy development process, given their role in economic growth and stability and alignment in values and ideology. There was also recognition that business stakeholders are well-equipped to provide a strong rationale for their preferred policy proposals:

“…business has a paved road rather than one they have to hoe themselves. They can get access to the Prime Minister and ministers…pretty much anytime they want to. So, if they’ve got a beef, they can be very influential….governments here just see business quite rightly as, you know, basically carrying the economy, and so if they’ve got a particular point of view, then they’re going to be able to make it…”

(I_07)

“…for better or worse, they run really good campaigns, and they put together, you know, ah, put together a war chest, put together champions, they put together um, the narrative and a, and a policy menu for government, and they, they run hard and they do it…”

(I_02)

NGOs can also play a role in the development of mitigation policy; however, it depends on the strategies they employ as to how influential they can be:

“...environment stakeholders if you like, to cast it a bit broader, are in my view most influential when they’re providing information-rich input. Ah, if they’re just sort of stating positions and lobbying, then it’s, it’s helpful and, you know, it helps in terms of the atmospherics around public policy and the realm of what’s possible, but in terms of informing a, actually information a policy process, it’s, it’s the…the more rigorous analytical stuff that’s helpful…”

(I_04)

“…the ones that are traditionally heard best are the ones that ah, have the strongest, most intellectually robust arguments ah, and cases, and that are not seeking out to embarrass, that are seeking out to persuade rather than embarrass.”

(I_07)

The role of experts in mitigation policy development has already been discussed in the section above. In addition to involvement through technical working groups or advisory panels, the importance of being perceived as objective and a good communicator can influence the level of input an expert has in the policy development process:

“…you’d be looking for somebody who’s, who’s, a scientist who’s policy neutral if you like, or as close to it as possible…if you’re an expert and you can craft an argument that’s of interest to, policy-makers and advisers, you know, in a highly, highly contested um, area such as climate change, you can find the, the policy-makers and advisers who think your point is relevant and the ministers ought to know, then you can be called in…experts can be heard if they can, if they can state, put their message in terms that are relevant to ah, the policy process…”

(I_07)

### 3.6. The Communication of Policy Decisions

Interviewees were asked about the drivers associated with the communication of climate policy, and whether health benefits and healthcare savings might be a useful communications frame in communicating policy decisions. Aside from the policy areas of vehicle emissions standards and energy efficiency, most interviewees argued that the use of health co-benefits to justify the implementation of mitigation policies would be limited. Most reasons provided focused on the same issues identified as barriers for the consideration of health co-benefits in the development of mitigation policies—issues around a lack of robust domestic data, the indirect nature of health co-benefits, as well as Australia’s current climate change narrative and mitigation policy approach:

“I think in general terms, it, it absolutely would help but I think, um, you need to look at it in the context of what, what policies you’re communicating. Um, I think with the current government’s policies as they are, you know, the reason they’re emphasizing things like agricultural productivity and energy productivity is because, as you know, it’s a sort of very direct action approach, and the, the communications are emphasizing that um, we can reduce emissions um, by taking direct action, and by taking direct action we’re actually helping farmers and um, and we’re helping businesses to continue to grow…I don’t think selling health um, outcome, or telling, talking about health outcome would work in the context of the government’s current policies and, and targets, cos I, I suspect um, any analysis of health benefits of action would probably say the targets aren’t high enough to achieve much benefit…”

(I_04)

## 4. Discussion

The results presented above indicate that health co-benefits currently play a minimal role in the development of national climate change mitigation policies in Australia. As the results outline, there are several factors that determine the extent to which multiple considerations, including health co-benefits, influence the mitigation policy development process. The case study above identifies that economic factors are one of, if not the most, significant consideration in the development of mitigation policy. This finding is similar to work undertaken by Baum and colleagues [33] on the social determinants of health, and aligns with the theoretical underpinnings of the political economy of health framework.

### 4.1. A Preoccupation with Economic Modeling

Several interviewees stated that economic modeling and analysis is seen as a crucial input that informs policy development, and has the potential to encourage the exclusion of certain considerations during mitigation policy development. One reason offered for the focus on modeling was that often the resulting numbers are considered objective, factual evidence, useful for justifying policy decisions:

“But the trouble is, as I said earlier, the trouble is, and, and this is really, really relevant in the Australian case, whenever you produce numbers, ministers think they’re facts. The only thing you can know about those number is they’re wrong, but ministers seem them as, as factual. You can stand in front of them and you can make, you know, an elegant and compelling argument ah, about why things should be done…in the interest of the Australian economy, the Australian people, the global commons, um, you know, the universe, love, death, everything, ah, but it will count for nothing against some joker who’s pulled out his phone ah, and uses the calculator and produces a list of numbers.”

(I_07)

In this way, quantitative inputs are prioritized over qualitative inputs in the policy development process. Some interviewees recognized the limitations of economic models, and the difficulty of addressing those limitations given the current institutional policy-making process, where the OBPR are required to approve a RIS and accompanying CBA prior to its submission to cabinet:

“The numbers of problems in the RIS process…I mean, mostly because it’s, you, you know, you’re often working in a social or in an energy, you end up, you know, some sort of policy area, and then you’ve got to put it in the right terms for the economists, then you’ve got to go and argue with the economists that their assumptions are not better than yours, and you’ve got to get them to approve it.”

(I_03)

A number of interviewees reinforced the contribution of modeling to the recent INDC target setting policy process. Ultimately, of the four target scenarios modeled—13%, 26%, 35%, and 45% absolute emission reductions compared with 2005 levels—the government settled on a target of 26%–28%:

“…the economic modeling that we did was about ah, the um, estimates of the economic cost to the economy of different um, different targets, with a kind of understanding that the whole point of this exercise was for Australia to, to play its fair share in um, in achieving global um, reductions in emissions, um and signing up to various um, if you like, commitments, like, um, the two degree commitment…but ultimately the um, the work was in, the work was designed to try and give a sense of how much, um, cost would be imposed on the domestic economy by signing up to different um, emissions reductions targets, and also to get a sense of what other countries were doing…that’s not completely straightforward because we’ve got different population growth from other countries, so it depends how you measure it…So um, we presented a lot of those sort of comparisons…”

(I_07)

“…my view is that way too much emphasis gets put on modeling outputs, um, especially…the 2030 target, it’s, it was all done last year, so you’re projecting fifteen years out, and um, the, the modeling um, you know, in terms of GDP impact, um, in 2030 associated with the four, five scenarios that were drawn from that modeling were all within the bounds of the margin of error anyway, so you can’t predict GDP fifteen years in advance with enough precision to…given, given the, the numbers that were coming out at the end of it, cos they were all, you know, 0.7, 0.4, and if it was, you know, if it was six compared to seventeen, then it’s meaningful…but there was um, in the target process, there was a lot of discussion about whether there should or shouldn’t be economic modeling and eventually there was ah, modeling, and a lot of the reason why there was discussion about should there be was um, because of the exact point of once you’re doing modeling and once there’s numbers, people just get fixated on those numbers and lose sight of um, the limitations of those numbers and the assumptions that are sitting behind them, um, and the lose sight of all the, the other considerations that sit around it…”

(I_04)

Bearing in mind the significant role of economic modeling and analysis to the policy development process, several interviewees suggested that numerous opportunities exist to increase the role of health co-benefits as a consideration in Australian climate change mitigation policy development.

### 4.2. Increasing the Role of Health Co-Benefits as a Consideration in the Development of Mitigation Policy

Firstly, there is an opportunity to integrate health more meaningfully into climate change mitigation policy in a comprehensive review of Australian climate change policy. A comprehensive review of climate change impacts on Australia has not been undertaken since the Garnaut Climate Change Review was commissioned by the Australian Labor Party and Australian state and territory governments in 2007, and as one interviewee lamented, the INDC target setting process had provided a platform but was not utilized:

“…so the INDC process was a missed opportunity essentially because what it, it, in theory those processes should provide an opportunity for national governments to assess what’s in their broader national interest…and I think the, the, one of the biggest failings in domestic policy, and it’s broader than health, is that we haven’t for a while um, attempted a systemic assessment of what climate change means for the systems which we um, need, whether they be health system or financial system or, you know, what the impact of global action is on our long-term prospects for our, our exports, for example…”

(PI_02)

The Coalition government, recently returned to power following a federal election in July 2016, has pledged to undertake a comprehensive review of Australian climate change policies in 2017. A number of interviewees identified this audit as a strategic time to raise the profile of health co-benefits and advocate for their inclusion in the mitigation policy development process.

In particular, areas of energy efficiency and sustainable transport were identified as key policy areas where health co-benefits are quantifiable and could be well received in Australia:

“…it’s only in relation to energy efficiency where you can claim, um, I think in Australia, where you can claim um, a, a carbon reduction measure as having a public health benefit. …A move to electrified transport would have a big impact on public health, because you’ve got…we’ve got all sorts of, of air pollution problems from ah, from combustion, internal combustion engines ah, in the cities.”

(I_07)

“…the benefit is those costs are avoided, um, and, and these are, you know, usually done on…the basis of ah, deaths and, and other kind of respiratory ill effects…and what the, the costs are there. …so we’ve been using it for a, a long time in the, the pollution space and…it’ll pull over into motor vehicle efficiency as well…”

(I_10)

The area of vehicle emissions is one where the Australian government appears to be genuinely considering health impacts. Following on from the establishment of a Ministerial Forum on Vehicle Emissions in late 2015, the Australian government released a Vehicle Emissions Discussion Paper in early 2016 [32]. The first paragraph of the Discussion Paper demonstrates a clear understanding of the link between the combustion of fossil fuels, health impacts, and climate change (p. 2):

“Emissions from motor vehicles can affect our health by polluting the air we breathe and can also contribute to climate change. To explore options to reduce the environmental and health impacts of emissions from motor vehicles, the Australian Government has established a Ministerial Forum to coordinate a whole of government approach to this important issue.”

Beyond the scheduled 2017 climate change policy review, the Council of Australian Governments (COAG) was suggested as an alternative avenue for promoting health co-benefits given the cross-jurisdictional nature of health in Australia’s federal system. While the Department of Health is responsible for the Environmental Health Standing Committee (enHealth), it has not recently provided any advice on health in climate change policy. enHealth’s Secretariat reports that a new national environmental health strategy is in the process of being prepared [34]. Interviewees also advised that a more cohesive awareness raising campaign was required to elevate the prioritization of health co-benefits as a consideration in the development of mitigation policy:

“You need, you need to marry, like the ideal world is you’d marry…a lobbying group like AMA…a policy advocate group on climate change…and the academic sector…it does need a level of credibility…and it also needs to be able to judge any policy that’s put forward against political pragmatism…”

(PI_02)

## 5. Conclusions

The case study presented here provides a level of insight into the role of health co-benefits in the development of Australian climate change mitigation policies. To do so, we explored the policy-making process; factors influencing the prioritization of multiple considerations; barriers and enablers to the consideration of health; the evidence base for policy; the role of external actors and stakeholders; and the communication of policy decisions. Results indicate that health co-benefits are not meaningfully considered in the development of mitigation policies in Australia. Explanations include a lack of local, robust data and champions both within and outside of government; the current Australian climate change narrative and a focus on domestic economic costs in mitigation policy development; as well as challenges associated with the long-term nature of health impacts and linking health co-benefits to climate change and fossil fuel use.

Based on responses from interviewees, a number of opportunities were identified for increasing the role of health co-benefits in the development of Australian climate change mitigation policies. Beyond addressing the acknowledged barriers, an upcoming government review of climate change policy in 2017 provides an opportunity for health co-benefits to be more meaningfully integrated into mitigation policy. This would require an environmental health champion to coordinate a cohesive and strategic policy campaign that speaks to the dominant climate change narrative within which policy-makers are currently embedded. Further, COAG was identified as a cross-jurisdictional avenue through which health co-benefits might be able to gain some political traction.

While the federal government is ultimately responsible for the development and implementation of climate change mitigation policies in order to meet international emissions reduction obligations, given Australian state and local governments are largely responsible for the development and implementation of health and adaptation policy, interviews with relevant state and local government employees may provide additional insight regarding the role of health co-benefits as a consideration in the development of climate change policy more broadly.

## Figures and Tables

**Table 1:** Key policy documents informing case study identified prior and during interviews.

Policy Document Title (Year of Publication)	Department Responsible for Publication
Emissions Reduction Fund White Paper (2014) [28]	Department of the Environment
United Nations Framework Convention on Climate Change (UNFCCC) Taskforce Final Report (2015) [29]	Department of the Prime Minister and Cabinet
National Climate Resilience and Adaptation Strategy (2015) [30]	Department of the Environment
National Energy Productivity Plan 2015–2030 (2015) [31]	Department of Industry, Innovation and Science
Vehicle Emissions Discussion Paper (2016) [32]	Department of Infrastructure and Regional Development

**Table 2:** Departments approached during the recruitment of interview participants.

Federal Government Department
Department of the Environment
Department of the Prime Minister and Cabinet (PM & C)
Department of Foreign Affairs and Trade (DFAT)
Department of Industry, Innovation and Science
Department of Infrastructure and Regional Development (DIRD)
Department of Health
